# Cambodia malaria indicator survey 2020: Implications for malaria elimination

**Published:** 2021-07-01

**Authors:** Soy T. Kheang, Ir Por, Siv Sovannaroth, Lek Dysoley, Huch Chea, Ly Po, Hala J. AlMossawi, Abu Al Imran, Neeraj Kak

**Affiliations:** 1 Health and Social Development (HSD), Cambodia; 2 National Institute of Public Health (NIPH), Cambodia; 3 National Malaria Control Program (CNM), Cambodia; 4 University Research Co., LLC, (URC) USA; 5 AQUITY Global Inc., (AGI) USA

## Abstract

**Background:**

Cambodia has made significant progress in controlling malaria in the past decade. It now aims to eliminate malaria from the country by 2025. It launched the Malaria Elimination Action Framework (MEAF 2016-2020) in 2015 with strong political commitment targeting appropriate interventions on high-risk populations, particularly mobile and migrant groups.

**Methods:**

In 2020, the household-level Cambodia Malaria Survey 2020 (CMS 2020) was conducted with the objective to assess the performance of malaria control activities using the indicators outlined in MEAF 2016-2020. The survey used a cross-sectional probability proportional to size approach drawing 4,000 households from 100 villages across the malaria-endemic districts of the country.

**Results:**

A total of 3,996 households with 17,415 inhabitants were interviewed. Of the surveyed households, 98.4% owned a long-lasting insecticide-treated bednet or hammock (LLIN/LLIHN). However, only 79.5% of these reported sleeping under a net the previous night, with only 45.7% sleeping under an insecticide treated net (ITN). Given that forest visitors are at the highest risk of getting malaria, the survey also targeted this group. Of the forest visitor respondents, 89.3% brought an ITN along and 88.9% reported to have used a net during their forest stay. About 10.8% of forest goers had received a forest kit for malaria prevention from mobile malaria workers the last time they went to the forest. Knowledge about mosquito repellents was high among forest goers (62.5%) but the actual use thereof during the last visit to the forest was low (22%). While awareness about malaria prevention with LLINs remained high among most respondents, knowledge about malaria diagnosis and treatment was not universal. Source of malaria knowledge and its treatment was usually from a household member, followed by a village malaria worker or a primary health care center staff. Of those who had fever during the previous two weeks, 93.6% sought advice or treatment outside the home, and the most commonly reported source for advice or treatment was private providers (39.4%) followed by health center/district hospital (31.3%).

**Conclusions:**

ITN distribution and other malaria prevention interventions have largely benefited the high-risk groups including the forest visitors. Comparing the CMS 2020 results with the 2017 CMS results, it is clear that forest visitors’ use of LLIN/LLIHN has improved considerably. However, more needs to be done to ensure forest visitors be protected either through using LLINs or repellents while working and staying in the forest areas. Also, given that sleeping under LLINs has decreased over the past several years among the at-risk populations, the programme will have to develop strategies to ensure that the communities do not lower their guard against malaria as cases further dwindle in malaria prone areas. Heightened awareness amongst the general population will be critical for eliminating malaria in Cambodia without any possibility of malaria re-emergence or re-establishment.

## Introduction

Considering the steady decline in malaria prevalence, Cambodia has set a goal to eliminate all forms of malaria by 2025. To achieve this goal, a National Strategic Plan to Eliminate Malaria by 2025 was endorsed by the Cambodian Prime Minister in 2011 [1]. Consequently, a Malaria Elimination Action Framework (MEAF) 2016-2020 was developed in 2015, outlining a stratified phased approach to achieving zero deaths and the elimination of *P. falciparum* by 2020 [2]. Recent programme data show that Cambodia continues to see a significant reduction in *P. falciparum* thus ensuring its route to become free of malaria by 2025. The number of reported malaria cases has declined from almost 100,000 in 2011 to 31,946 in 2019 (of which 85% were caused by *P. vivax*) with a national annual parasite incidence (API) of 3.46 per 1,000 population [3]. Given the programme’s success so far, Cambodia launched the Malaria Elimination Action Framework (MEAF) 2021-2025 in 2020, shifting from a phased approach (elimination, transitional, and high burden) to a whole-country elimination strategy [4]. Under the new MEAF, the 6,422 villages in endemic operational districts (ODs) were stratified into six categories (no-, low-, medium-, high-, and highest risk) based on village-level API for 2018 and 2019, and the percentage of forest cover within a 3 km radius of the centre of each village. Key interventions and their implementation modality will be adapted according to this stratification with the higher the risk the more interventions will be employed, with higher frequency.

The MEAF 2016-2020 included a number of key indicators to track the progress on specific malaria elimination objectives. While most of these indicators rely on routinely collected data through the Malaria Information System (MIS), some indicators, mainly those related to the support from the Global Fund to fight HIV/ AIDS, Tuberculosis and Malaria (GFATM), require population-based data collected through national household, facility, and community surveys. Since the start of the Cambodia malaria indicator surveys, namely Cambodia Malaria Surveys (CMSs), data on knowledge, attitude and practices have been collected using household and facility-based surveys. Since the first survey in 2004, follow up CMS were conducted in 2007, 2010, 2013 and 2017. Measurement of malaria prevalence was conducted until CMS 2013 [5]. Due to the sample size requirements, measurement of malaria prevalence was excluded from the CMS 2017 [6].

## Survey Objectives

The main objective of the CMS 2020 was to assess the performance of malaria control activities in endemic regions of Cambodia, using indicators outlined in the MEAF 2016-2020. The survey also assessed the perceptions about mosquito repellents and other interventions, including mass drug administration [7,8], among the forest visitors.

## Methodology

### Sampling and sample size

**Figure 1:**
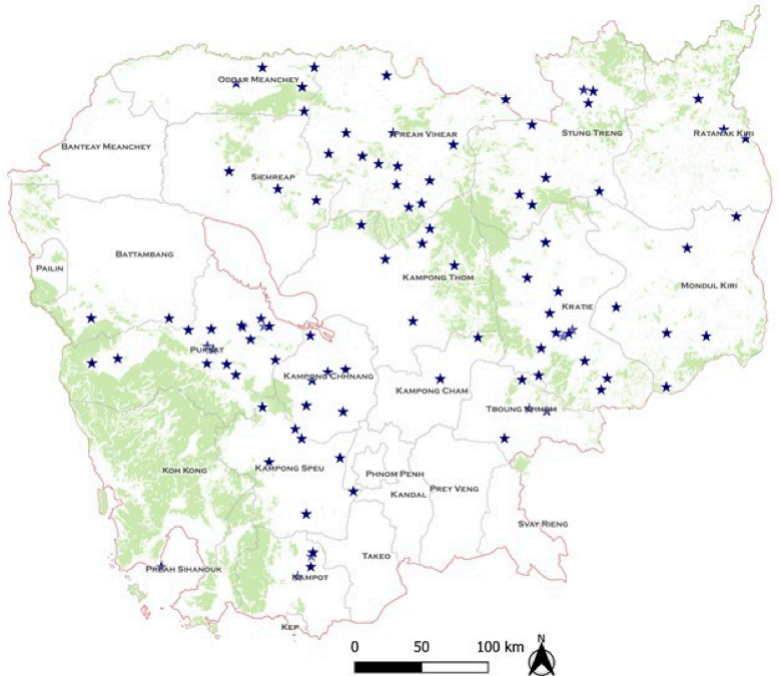
Map of Cambodia with the 100 villages selected for the survey.

The CMS 2020 targeted 1,412 malaria-endemic villages with an estimated population of 1,151,578 or 255,906 households. These villages are distributed across the catchment areas of 260 health centres in 44 operational districts and 19 provinces. The survey used a probability proportional to size with a two-stage sampling process. First, 100 villages (clusters) were randomly selected from the sampling frame. Figure 1 shows the map of Cambodia with the 100 selected villages. The second phase included the random selection of 40 householders in each selected village.

### Data collection and analysis

#### Data collection tool

The CMS-2020 data was collected by trained interviewers using structured household and forest visitor questionnaires. Data was collected on handheld tablets using pre-programmed structured data entry software. The household questionnaire was administered to the female head of the household whereas forest goers’ questionnaire was administered to the individual forest visitors from the household [9]. The household questionnaire was developed based on previous rounds of the CMS surveys. The forest visitors’ questionnaire was developed based on the questionnaire of the Cambodia Mobile and Migrant Population Survey 2017 [10] and the CMS 2017 questionnaire with additional questions added on the perceptions about new potential malaria interventions. The forest visitor questionnaire also included questions on the time and duration of the visit to the forest, accompanying preventive behaviours and practices, including use of Long-Lasting Insecticidal Hammock Net (LLIHN), mosquito repellents and taking antimalarial drugs, and their perception about mass drug administration.

#### Field data collection

Data was collected through GPS-enabled tablets that automatically uploaded data into a cloud-based database server. A relational database was designed to capture all critical survey variables. Automatic data checking was introduced to ensure real-time data cleaning. The central data management team also cross-checked the data and implemented data cleaning and validation on a regular basis.

#### Data analysis

Data were analysed using Stata SE. Key coverage indicator values were computed in percentage with 95% confidence intervals (CI). Data were analysed by gender, geographic location, API levels as a risk stratification (level 3, 4, and 5), as well as other demographic characteristics and household socio-economic status. The household socio-economic status (poverty index using quintiles) was computed based on the household ownership of durable assets and access to water, sanitation and essential social services [11]. The study used Chi-square tests to compare proportions between population groups (e.g., the poorest quintile versus richest quintile) and significance was determined at the 5% level (P<0.05). Means of normally distributed data between population groups was compared using Independent-Sample t-tests and a non-parametric test (Mann-Whitney) was applied for skewed data.

### Ethical considerations

The study protocol was reviewed and approved by the National Ethics Committee for Health Research in Cambodia (Reference number: 155/NECHR dated 29 June 2020). The data collection team followed strict ethical norms and sought informed consent from each survey respondent.

## Results

In total, the study covered 3,996 households with 17,415 individuals, distributed across 100 villages, 95 communes, 94 health centre catchment areas, 54 districts, 33 operational districts, and 16 provinces. Of the 17,415 individuals, 994 (5.7%) reported to have ever visited the forest and slept overnight there (forest visitors).

### Socio-demographic characteristics

The survey respondents included 48.6% males and 51.4% females. A significant number of respondents had never attended school (29.1%), whereas 41.3% had some primary education, 10.1% with completed primary education, 14.8% with some secondary, 3.2% with complete secondary education, and only 1.1% had higher education. Almost all of the respondents lived in the communities (99.4%) and only 0.6% of them were temporary visitors. Over 94% of them reported to have slept in the house the night before the survey, and only 5.9% did not do so. The reasons for not sleeping in the house the night before the survey were: work in another province or in a city (30.7%), work in *chamkar*/plantation (23.7%), visit relatives/ friends or going for holidays (20%) or work in the forest (12.2%).

The mean household size was 4.36. Most (81%) of the households were headed by men. The current main occupation of the household heads was farming (74.4%), followed by market vender (8.1%), civil servant (3.7%), fishing (2.3%) or manual labourer (1.9%). The occupation that provided the primary source of income for the households was predominantly farming (61.7%), followed by market vender (10.6%), manual labourer (6%) and civil servant (4.5%). Timbering/logging is a fifth (3.6%) source of income. Of the surveyed households, 89.1% reported to have at least one mobile phone, nearly 85.8% having a motorbike and 64.4% having access to electricity. The median number of households sleeping spaces was 2 (range 1-7). Only 2.9% of the households reported to have used metal or plastic screens on windows to keep mosquitoes out, while 47.8% used other methods (e.g., sprays, coils or repellents). The median household expenditure for these methods in the past month was US$ 3.0.

### Net ownership and use

**Figure 2:**
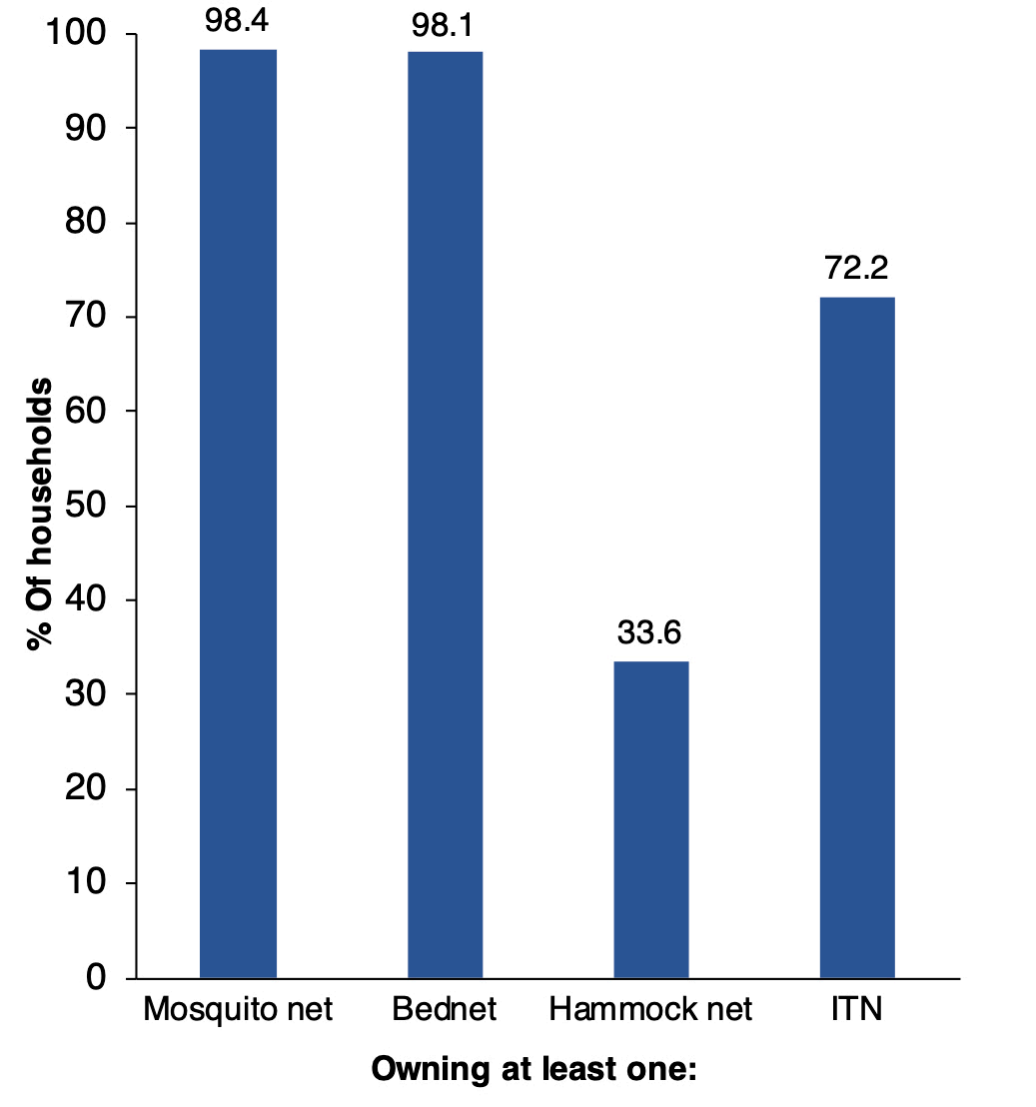
Percentage of households that owned at least one mosquito net by type of net (n=3,996).

As shown in Figure 2, 98.4% of the survey households owned at least one mosquito net. Similarly, 98.1% of them owned a mosquito bednet but only 33.6% owned a hammock net. Of the total households, 72.2% owned an insecticide treated net (ITN) – be it an LLIN or Long-Lasting Insecticidal Hammock Net (LLIHN).

Among households having mosquito nets, on average, each owned approximately 3 mosquito nets (ranging from 1-12), including 2 bednets and one hammock net, and 2 ITNs (ranging from 1-10). Of all the mosquito nets owned by the households (11,581), 65% are ITNs, including 63% LLINs and 2% other ITNs, which could not be identified as LLINs. Conventional nets accounted for 31% of the nets. The survey also found that the largest source of nets (LLINs) is the government (54%), followed by shops/markets (18%), itinerant sellers (13%), NGOs (10%); the rest reported to have received it from other sources (5%).

**Figure 3:**
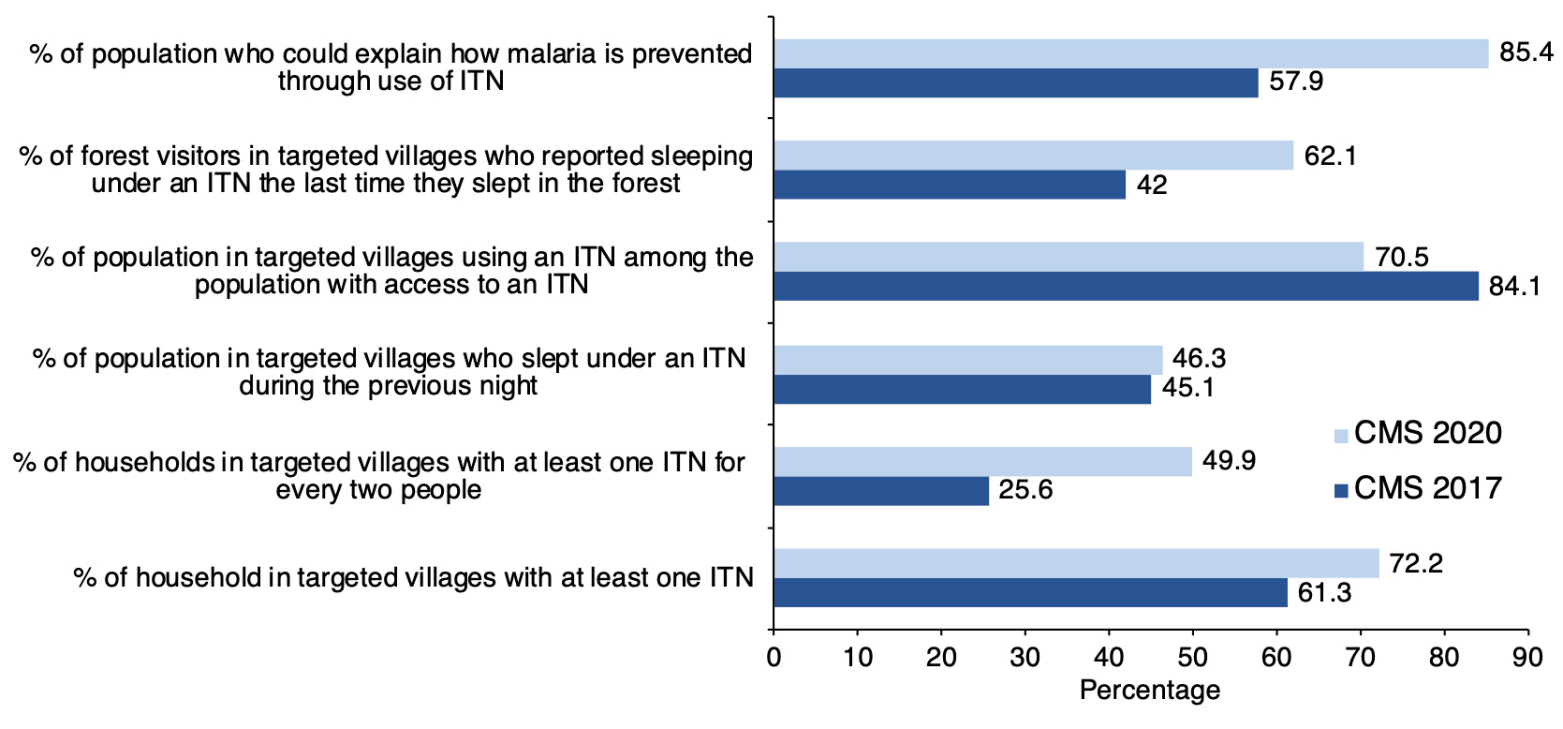
Changes in key malaria elimination indicators between the CMS 2017 and CMS 2020 surveys.

Figure 3 provides an overview of changes in key malaria elimination indicators between the CMS 2017 and CMS 2020 surveys. Although the ownership of ITNs increased from 61% (in CMS 2017) to 72% (CMS 2020), there has been a larger increase in the percentage of households with at least one ITN per two people; from 26% to 50% over the same period. However, it is clear from the CMS 2020 survey that only 70% of households reported to have slept under an ITN the previous night compared to 84% reported in the CMS 2017 survey. Among the forest visitors, significant improvements were noticed in the use of ITNs between the two surveys, showing an increase from 42% in 2017 to 62% in 2020. Also, there was an overall improvement in the knowledge about how to prevent malaria by ITNs which increased from 57% to 85%.

### Malaria prevention knowledge and practices among forest visitors

Of the 787 interviewed forest visitors in CMS 2020, 11% reported to have received a forest kit (e.g., from a mobile malaria worker) the last time they went to the forest. While 63% of forest visitors reported that mosquito repellents can prevent them from getting malaria during their stay in the forest, only 22% reported to have used mosquito repellents during their last stay in the forest (Table 1). Many of them (77.2%) did not use such preventive method, mainly because there was no repellent available for them to use (78.5%). The majority of mosquito repellents used (77.9%) was ‘bought from the market, and 12.2% was given as part of forest kit. Only 5.2% was shared by another forest visitor.

**Table 1:** Utilisation of mosquito repellents by forest visitors.

Variables	Number	%
Use of repellents by forest visitors during their stay in the forest	(n=787)	
Yes	172	21.9
No	608	77.2
Not sure	7	0.9
Sources of repellents that the forest visitors used	(n=172)	
Bought from market	134	77.9
Was given as part of forest kit	21	12.2
Shared by another forest visitor	9	5.2
Other sources	8	4.7
Main reasons for forest visitors not using repellents	(n=604)	
No repellent available	474	78.5
I don’t think it helps	39	6.5
Use hammock net	22	3.6
No money to buy it	12	2.0
Forgot to take it from home	10	1.7
No habit to use it	9	1.5
Other reasons	19	3.1
Don’t know	19	3.1

Besides the use of mosquito net and/or repellent, a large proportion of forest visitors reported other malaria preventive measures such as burning leaves (50.2%), wearing long clothes (23.9%) or burning a mosquito coil (21.3%).

CMS 2020 also asked forest visitors questions about attitudes and practices in relation to the use of oral medicines to prevent malaria while in working in forested areas. While 450 (57.2%) of the forest visitors ever thought to bring some oral medicines along when going to the forest, only 31.8% of them said that they did bring such oral medicines along with them the last time they went to the forest and 66.7% others did not do so. The main reasons for forest visitors who did not bring oral medicines along with them are mainly ‘no oral medicines to bring’ (47%) and ‘not necessary to bring’ (42.5%).

The medicines they brought along with them to the forest were predominantly paracetamol (81.2%) and unspecified fever medicines (8.4%), followed by anti-malarial medicines (e.g., artesunate-mefloquine), which accounts for 6.4% of the forest visitors. Without access barriers, more forest visitors would bring oral medicines along with them to the forest. The oral medicines they wished to bring are still first paracetamol (73.6%), followed by antimalarial medicines (16.9%).

In response to the question ‘if someone gave you antimalarial medicines (oral tablets) and requested you to take them when going to the forest, would you agree to take them as advised?’, 507 (64.4%) said yes while 234 (29.7%) said no and 46 (5.8%) others answered, ‘not sure’. Only 53 (6.7%) of the forest visitors reported to have been sick while away from home or staying in the forest. Among them, 39.6% sought treatment from village malaria workers/ mobile malaria workers (VMW/MMW) and 24.5% did not seek any treatment. 13.2% sought treatment from a private health provider and only 7.5% did so at health centre or former district hospital.

### Malaria knowledge, attitude, and practices

Knowledge of malaria was assessed by asking questions to adult household respondents, commonly the head of households. A total of 3,938 (98.5%) of the 3,996 surveyed households reported to have ever heard of malaria. The 58 households (1.5%) who reported to have never heard of malaria were not asked further knowledge-associated questions. Only 12.4% and 12.3% of the household respondents correctly answered about RDT/ dipstick and blood test (slide) to confirm if someone in the household has malaria. A much larger proportion of household respondents wrongly referred to doctor’s examination, symptoms, and previous experience.

## Discussion

The CMS 2020 results show that household ownership of mosquito nets, in particular ITNs, is high and significantly higher than what was reported in the CMS 2017. This clearly demonstrates that the programmatic interventions including LLIN/ LLIHN distribution have resulted in more households with ITNs. However, the data also show that there has not been a corresponding increase in the use of ITNs. The percentage of the population with access to an ITN sleeping under an ITN the previous night was high at 71% but significantly lower than the CMS 2017 finding (84%). Like net ownership, the percentage of the population sleeping under an ITN also declines by household wealth and increases by level of risk. The stagnation of ITN utilisation coverage among villagers may reflect changing risk perception given a significant decline in malaria prevalence (reflected by malaria case load) in the communities.

Nevertheless, for forest visitors for whom the stay in the forest still poses a high risk, the ITN utilisation rate is relatively high and significantly higher than what found in CMS 2017. Of the 787 interviewed forest visitors, 89% reported to have used a mosquito net of any type during their stay in the forest, mainly with a hammock net (54%) and 62% of them did so with an ITN, compared with 42% in CMS 2017. However, more work needs to be done to ensure that all forest goers utilise ITN while sleeping in the forest. The programme also will need to look at the role of either mass drug administration or repellents among the forest goers.

86% Of the respondents considered malaria to be a major threat for households and the community among others diseases such as including dengue or COVID-19. As found in CMS 2017 and Cambodia Mobile Migrant Population Survey 2017 [10,11], nearly all the household respondents (99%) reported to have heard of malaria, and their knowledge about malaria (including signs and symptoms of simple and severe malaria, diagnosis, transmission, and prevention) is generally good. Fever and chills which are the most common symptoms of malaria were reported by 90% and 83% of the respondents, respectively. Similarly, almost all signs and symptoms of severe malaria were reported by a large proportion of respondents, with 81% for high fever/ temperature, 32% for unconsciousness and 30% for convulsions. Knowledge on malaria prevention is also good, with 92% of household respondents reporting sleeping under a mosquito net to prevent malaria. Over 85% of the household respondents could explain how malaria is prevented by using an ITN, which is significantly higher than the results of CMS 2017 (58%). However, misconception remains because 13% of respondents reported boiling water to prevent malaria. Knowledge on malaria diagnosis (means to confirm someone having malaria) is not so good. RDT/ dipstick and blood test (slide) to confirm malaria diagnosis are reported equally by 12% of household respondents, while others referred to doctor’s examination (31%), symptoms (23%) and previous experience (21%) with malaria diagnosis. Like the findings of CMS 2017 and the Cambodia Mobile Migrant Population Survey 2017 [12,13], almost all respondents could correctly identify at least one resistance/relapsing effect because of not taking all prescribed antimalarial medicines or taking them fewer days than recommended with ‘patient does not recover’ and ‘patient gets sick again’ being the two most reported.

Only 2.4% of the surveyed individuals reported to have been ill with fever in the past two weeks prior to the survey. This is much lower than the 2017 result (8.6%). This could be partly linked to the effect of COVID-19 prevention efforts on cold and flu and the declining malaria incidence. Of the individuals with fever in the past two weeks, 93.6% sought advice or treatment outside the home for the fever, compared with 90.5% in 2017. The most reported source of the advice/ treatment is private provider (39.4%), followed by health centre/former district hospital (31.3%). Only 3.3% of those seeking advice or treatment reported to a VMW as a first point of consultation for fever amongst villagers, and this figure increased to 15.8% for those with malaria related fever (*krun janh/krun lors*). Among forest visitors who were sick while away from home or staying in the forest, consulting a VMW was much higher at 40%. The CMS 2017 found 22.8% of the surveyed population which also include non-targeted population [7]. This suggests that the choice of providers for malaria advice/treatment among population in targeted villages is mainly health centres and former district hospitals as the first choice, followed by private providers, and VMW is mainly chosen for malaria related advice or treatment and mainly for forest visitors. The current (perceived) risk of malaria transmission in the targeted villages is very low, and only 4.6% of the reported fever was malaria related fever. This suggests that broadly defined fever is no longer considered a proxy of malaria infection. Other reasons could also have contributed to this low use of VMW as a first point of consultation for fever among population in targeted villages: lack of awareness of VMW (by 19% of the respondents who said they did not know there was VMW in their village), and lack of confidence in or dissatisfaction with VMW performance (health centres/ referral hospitals/provincial hospitals/pharmacy is seen to be better than VMW); VMW did not standby at home, and did not have enough medicine and equipment (18%).

Like many other cross-sectional studies [14,15], findings from this snap-shot survey may not entirely reflect the actual situation throughout the year. Moreover, self-reported data could be subject to recall bias, especially with one respondent for all members in the household, except for forest visitors. For forest visitors, the recall period of three months for the visit to the forest and the ambiguous definition of forest in some places may make the data collection challenging. Moreover, some forest visitors did not sleep overnight in the forest (but did pass by the forest during night-time), and thus, were not included in this study. This should be considered in future studies on forest visitors. We also acknowledge that the way questions about knowledge on malaria risk and prevention is structured, with a majority of correct answers placed on the top may not be optimal and could be subject to social desirability bias.

## Conclusions

Key findings of this survey suggest that the efforts in distribution of LLINs/LLIHNs by the national programme and partners over the last few years have benefitted the population at higher risk of malaria, resulting in increased ownership of ITNs in malaria-endemic areas. The utilisation of ITNs among forest visitors also increased significantly since the 2017 CMS survey. The findings also suggest that a majority of forest visitors have positive attitudes towards mosquito repellents, and potential intervention such as chemoprevention using antimalarials.

Nevertheless, nearly two-fifth of forest visitors still did not sleep under an ITN during their stay in the forest, mainly because of access problems and perceived need. Moreover, anecdotal evidence suggests that many forest visitors pass by and stay in the forest (have exposure to malaria infection) but do not sleep overnight in the forest, and thus, cannot sleep under ITN and do not benefit from this intervention. At the same time, the utilisation rate of ITNs among villagers remains relatively low, even lower than the previous years, which also is partly related to access problems and decreasing (perceived) risk of malaria transmission in the targeted villages. As pointed out by other studies, there is a need to improve malaria prevention practices among both the general as well as forest goer populations [16].

Since malaria infection is currently mainly happening among forest dwellers, further efforts in improving the utilisation of ITNs by this population group as a malaria prevention measure among others is necessary for malaria elimination. Such efforts should focus on increasing their access to LLIHNs through more effective LLIHN distribution strategies accompanied by relevant educational messages. In addition, other malaria preventive interventions that have potential in complementing ITN such as mosquito repellents and chemoprevention (mass drug administration or targeted drug administration) should be considered. Mosquito repellents could be complementary to ITN use, especially for forest visitors who do not really sleep in the forest or those who sleep but not fully during the whole night. However, introduction and scaling up of these interventions should be done with caution and further in-depth studies on this should be considered to inform nationwide scaling up of these interventions.

While the utilization of ITNs among villagers is low, efforts to improve such utilisation in all targeted villages (as defined by the current list of villages targeted for LLIN/LLIHN distribution), including LLIN distribution and associated information, education and communications, may not be necessary. Such efforts should focus more on relatively high-risk villages based on an updated list of targeted villages stratified by risk level.

Anecdotal evidence about forest visitors who passed by and stayed in the forest (thus being exposed to malaria infection) but do not sleep overnight in the forest deserves further investigation to assess the extent of the problem and possibly look for solutions.

Finally, the very low risk of malaria transmission in the targeted villages and low proportion of malaria-related fever among all the reported fever suggests that broadly defined fever is not a relevant proxy of malaria infection anymore, except for forest dwellers.
